# Introducing the addictive daydreaming scale: development and Polish validation of the ADS-20 and ADS-5

**DOI:** 10.3389/fpsyt.2025.1702416

**Published:** 2025-12-15

**Authors:** Igor J. Pietkiewicz, Anna M. Hełka, Radosław Tomalski

**Affiliations:** 1 Research Centre for Trauma and Dissociation, Ignatianum University in Cracow, Cracow, Poland; 2 Institute of Psychology, SWPS University, Warsaw, Poland

**Keywords:** maladaptive daydreaming, addictive daydreaming, psychometric validation, scale development, screening tool, compulsive fantasizing

## Abstract

**Background and aims:**

Maladaptive daydreaming (MD) is a pattern of excessive, compulsive fantasizing associated with functional impairment. While its classification remains debated, increasing evidence suggests that it may be understood as an addictive form of emotion regulation. This study aimed to develop and validate a new self-report tool, grounded in the behavioral addiction framework, for screening and assessing the severity of MD, and to examine its potential clinical utility.

**Methods:**

A mixed-clinical (N = 182) and non-clinical (N = 231) sample completed the Addictive Daydreaming Scale (ADS) in two forms: a 20-item full version (ADS-20) and a 5-item screening test (ADS-5), together with established measures of MD, quality of life, and self-rated impact of daydreaming.

**Results:**

Psychometric analyses confirmed that both the ADS-20 and ADS-5 demonstrated high reliability and strong content, criterion, and construct validity. ADS scores were more strongly correlated with both the psychological and social domains of quality of life than Maladaptive Daydreaming Scale scores, supporting its sensitivity to functional impairment. Cut-off scores of ≥42 (ADS-20) and ≥8 (ADS-5) showed good accuracy for identifying clinically significant cases.

**Conclusions:**

The ADS-20 and ADS-5 are reliable, valid instruments for identifying and assessing addictive features of maladaptive daydreaming. Their use may support improved clinical detection, assessing the severity of MD and help conceptualize it within the behavioral addictions framework.

## Introduction

Fantasy plays a fundamental role in psychological development and everyday functioning. For example, children use fantasy or daydreaming to internalize caregiver figures, manage separation anxiety, or support cognitive growth (1). Fantasy can also aid role exploration and enhance planning abilities, including the capacity to set and achieve goals (2–4). Freud (5) emphasized its function in resolving intra-psychic conflicts and compensating for unmet emotional needs. Furthermore, daydreaming has been recognized as a coping strategy for trauma, allowing individuals to divert their attention, escape distressing experiences, alter their perception of such experiences, and evoke positive emotions (6–8).

However, in some individuals, daydreaming transitions from a flexible coping strategy into a rigid, compulsive, and impairing pattern. Initially providing reward and relief from negative affect—such as loneliness, shame, or boredom—it may escalate, consume increasing amounts of time, and replace healthier coping strategies (Soffer-Dudek et al., 2025). This maladaptive pattern can result in significant distress and functional impairment, including social withdrawal, academic underachievement, and impaired occupational functioning. Somer (9) coined the term *maladaptive daydreaming* (MD) to describe this phenomenon, defining it as “an extensive fantasy activity that replaces human interaction and/or interferes with academic, interpersonal, or vocational functioning.”

MD is increasingly acknowledged as a clinically relevant condition associated with significant impairment, though its precise diagnostic status remains debated. It is often hidden due to shame and internal conflict, with many affected individuals struggling to disclose the behavior (7, 10). Although MD may begin as a coping mechanism for psychological distress, it often intensifies the very difficulties it seeks to alleviate over time. Comorbidity with personality disorders, anxiety, depression, and trauma-related disorders is frequently reported (11). A growing body of research has documented its prevalence in both general and clinical populations, with higher rates among students and individuals with psychiatric conditions.

Recent epidemiological research suggests that maladaptive daydreaming (MD), although still under-recognized in clinical settings, is not particularly rare. For example, a systematic community survey conducted in Israel found that 4.2% of the sample (N = 1,023) screened above the threshold on the Maladaptive Daydreaming Scale, with even higher rates observed among young adults and students. Following clinical interviews, the estimated point prevalence in the Israeli-Jewish population was approximately 2.5% (12). An Italian online survey conducted during the COVID-19 lockdown identified 17.2% of participants (N = 6,277) as likely to have MD (13). Another study focusing on medical students in Sudan found that roughly one-third (approximately 34%) self-reported symptoms consistent with MD (14). These findings highlight that MD is not confined to clinical populations and appears at notable levels in broader community samples, though reported prevalence varies depending on recruitment methods and assessment tools.

In terms of comorbidity, MD rarely presents in isolation, though only a limited number of studies have investigated its occurrence in mixed-clinical populations and specific diagnostic groups (11). Meta-analytic data demonstrate strong associations between MD and a wide range of psychiatric symptoms and traits (15). Pietkiewicz et al. (16) found that over 66% of 51 patients diagnosed with narcissistic personality disorder (NPD), using the SCID-5-PD, exceeded the clinical cutoff on a self-report measure of MD. West, Somer, and Eigsti (17) investigated 235 participants who self-reported having received an autism spectrum disorder (ASD) diagnosis from a clinical psychologist, pediatrician, or qualified team; 43% of them also met criteria for MD.

Soffer-Dudek, Aquarone, and Somer (18) explored MD in 67 UK-based patients diagnosed with dissociative identity disorder (DID) via the SCID-D and found that 25% met diagnostic criteria for MD during a structured clinical interview. Similarly, Theodor-Katz et al. (19) investigated MD in a sample of 83 individuals diagnosed with attention-deficit/hyperactivity disorder (ADHD). Among those who scored above the clinical cutoff on an MD screening measure, 20.5% were confirmed to have MD through structured diagnostic interviews. In a more recent study, however, 34 out of 72 ADHD patients (47%) also met diagnostic criteria for MD (20). In a clinical sample of 100 Israeli Arab men recovering from substance use disorder (SUD), 16% scored above the threshold for MD (8).

**Figure 1:**
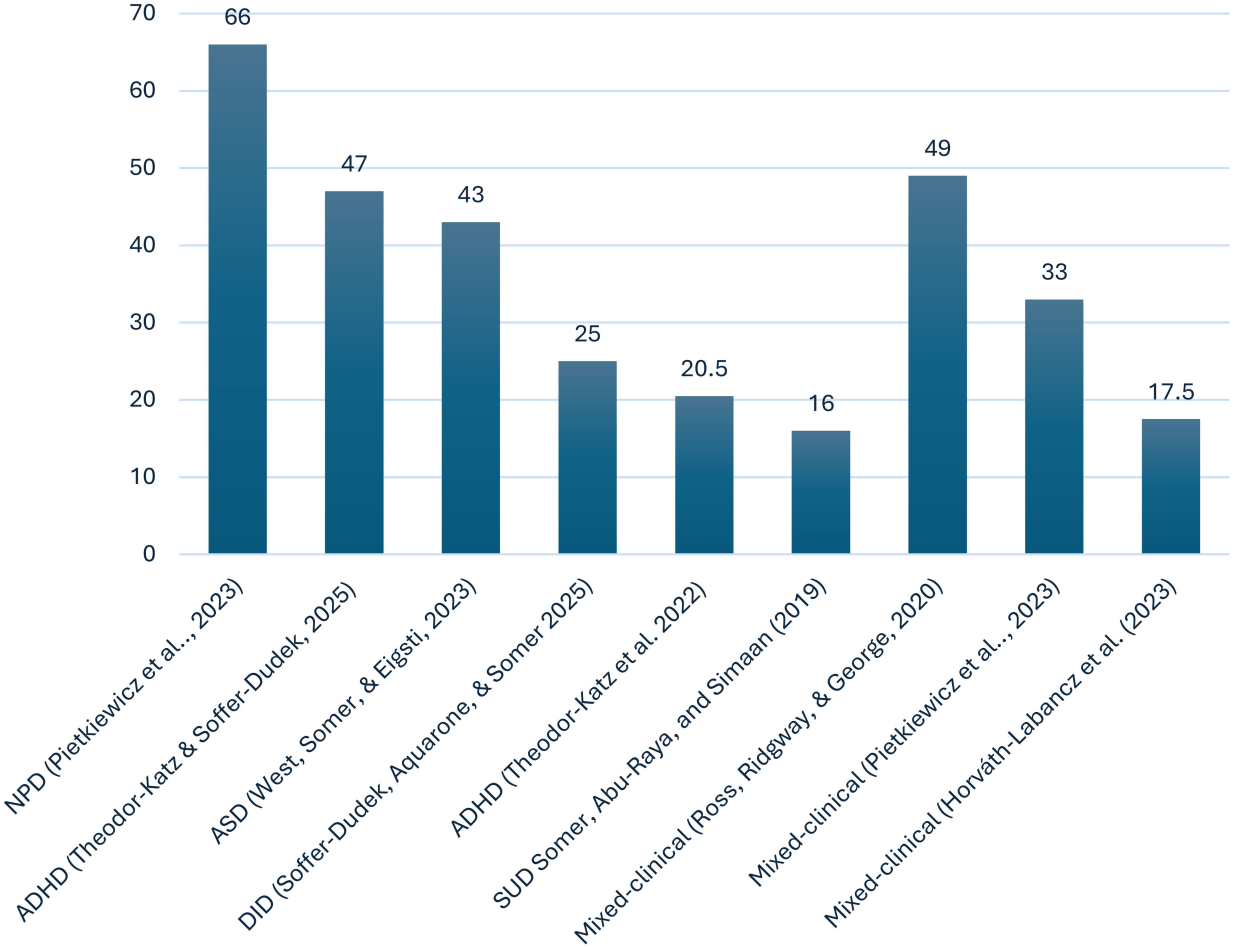
Maladaptive daydreaming in patient populations: confirmed and self-reported diagnoses.

The prevalence of MD in mixed psychiatric groups differs depending on the sample and methodology used. In a Hungarian mixed-clinical sample (N = 239) studied by Horváth‐Labancz et al. (21), nearly 17.5% reported MD. In Polish studies, between 33% and 40% of patients exceeded the cutoff point for MD (16, 22). Another noteworthy investigation involving 100 patients from a private psychiatric facility specializing in trauma-related disorders found that 49% met criteria for MD when assessed via the Structured Clinical Interview for Maladaptive Daydreaming. Interestingly, despite this relatively high prevalence, approximately half of the participants scored below the recommended clinical threshold on a widely used screening measure (23). This discrepancy raises important questions about the sensitivity and ecological validity of current screening tools for MD, particularly in populations characterized by complex trauma histories (see **Figure 1**).

### Rethinking maladaptive daydreaming – classification controversies

The classification of MD remains a topic of ongoing debate. Some researchers argue that it should be conceptualized as a dissociative disorder for several reasons. Firstly, Soffer-Dudek et al. (2025) point to daydreamers’ reduced responsiveness to external stimuli and a sense of disconnection from their subjective experience as hallmarks of dissociation. These characteristics align with the DSM-5’s definition of dissociative disorders, which involves disruptions in the normally integrated functions of consciousness, memory, identity, or perception of the environment.

While MD can involve intense fantasy absorption, a narrowed attentional focus, and blurred boundaries between reality and imagination, these features do not constitute its core problem. Instead, MD is characterized by a compulsive, addictive engagement in daydreaming that leverages one’s capacity for absorption. In contrast, in depersonalization/derealization disorder, dissociative symptoms are central to the condition, whereas in MD, absorption and alterations in consciousness merely accompany the fantasizing activity.

A second argument supporting the dissociative model of MD is the reported comorbidity (Soffer-Dudek et al. 2025). In the study by Soffer-Dudek, Aquarone, and Somer (18), 25% of patients diagnosed with dissociative identity disorder (DID) reported symptoms of MD during structured clinical interviews. Similar rates were observed in 23% of trauma survivors at risk for dissociative disorders, as studied by Ross, Ridgway, and George (23). While these results suggest some overlap, they cannot substantiate the claim that MD is a dissociative disorder. Notably, among all clinical populations studied to date, the highest levels of MD (66%) were found in individuals diagnosed with narcissistic personality disorder (16), which is not typically classified within the dissociative spectrum.

Similarly, daydreamers’ elevated scores on the Dissociative Experiences Scale (DES-II) and its subscales, such as depersonalization and derealization (24–26), cannot be regarded as indications of dissociative nature of MD. High scores on this instrument are also observed in individuals with anxiety, depression, personality disorders, and trauma histories (27).

Interestingly, Soffer-Dudek et al. (2025) state that MD “is addictive and structured, substituting other adaptive coping strategies,” and that it “could be viewed as a behavioral addiction, but there is no current addiction diagnosis suited for it.” Conceptualizing MD as a dissociative disorder shifts the focus away from what is truly maladaptive in MD: the addictive nature of the mental activity. Pietkiewicz et al. (7) have argued that excessive and compulsive daydreaming meets criteria for a behavioral addiction and should be conceptualized as such.

The behavioral addiction model offers a more comprehensive framework for understanding MD, particularly in its more severe manifestations. Griffiths (28) outlined six core components of behavioral addictions: (1) salience – the behavior dominates thinking and behavior; (2) mood modification – engagement produces emotional relief or pleasure; (3) tolerance – increasing amounts are required for the same effect; (4) withdrawal – discomfort when the behavior is restricted; (5) conflict – interpersonal or intrapsychic struggles due to the behavior; and (6) relapse – reversion to problematic behavior after attempts to reduce or stop. Many individuals with MD report precisely these features (7, 29).

Although only gambling disorder and gaming disorder are currently included in the ICD-11 as disorders due to addictive behaviors (29), other candidate behaviors – such as problematic pornography use, compulsive buying, and social network addiction – are being actively researched (30). To avoid premature medicalization, Brand et al. (31) recommend three meta-level criteria for including new behavioral addictions under the ICD-11 category “other specified disorders due to addictive behaviors”: 1) Clinical relevance, demonstrated by significant impairment in daily life; 2) Theoretical embedding, with the behavior explainable by existing addiction models; 3) Empirical support, including psychological and neurobiological evidence of addiction processes.

These criteria aim to distinguish true behavioral addictions from transient, context-bound habits. Similarly, Billieux et al. (32) stress the need to establish functional impairment and behavioral stability over time before labeling a behavior as addictive. Many excessive behaviors remit spontaneously; only those that persist, escalate, and cause dysfunction warrant formal classification.

Further support for the behavioral addiction framework comes from distinctions between addictive and impulsive disorders. As Rumpf and Montag (33) argue, compulsive sexual behavior disorder, now classified as an impulse control disorder in ICD-11, may eventually be reclassified as an addictive behavior, much like gambling disorder. While impulsive acts are often reactive and aimed at reducing tension, addictions involve reward-driven, habitual patterns that intensify over time and result in functional decline. MD shares more with the latter: it is typically immersive, sustained, and resistant to voluntary control, often driven by a craving for reward or emotional regulation rather than immediate impulse.

In line with the recommendations of Brand et al. (31), who suggested that candidate behavioral addictions in ICD-11 should be evaluated against the diagnostic framework for Gaming Disorder, we have formulated provisional criteria for daydreaming disorder, reflecting an addiction model: 1) Impaired control over daydreaming; 2) Increasing priority given to daydreaming over other life activities; 3) Continued or escalated use despite distress or impairment. These criteria provide a foundation for operationalizing the concept of addictive daydreaming and differentiating between mild and severe cases (see **Appendix 1**). Future research should focus on developing diagnostic interviews to confirm these criteria in clinical settings and exploring neurobiological markers that may support an addiction model.

### Measuring addictive daydreaming

To advance clinical understanding and assessment of addictive daydreaming, there is a need for instruments that capture its defining characteristics within the behavioral addiction framework. A brief screening tool would aid clinicians in identifying the syndrome in routine practice, while a more comprehensive measure—capturing both frequency and negative consequences—would support monitoring treatment progress and assessing intervention outcomes. Such tools could also advance large-scale epidemiological studies, improve diagnostic accuracy, and inform both clinical practice and theoretical models. Furthermore, they would facilitate research into the neurobiological and psychological mechanisms underlying this compulsive form of inner experience.

In the present study, MD is conceptualized as a harmful pattern of emotion regulation that—under certain conditions—may evolve into a full-fledged behavioral addiction. While the term MD remains widely used and clinically meaningful, we propose that it be understood within the framework of behavioral addiction as part of a continuum of severity. Specifically, MD may describe mild cases of daydreaming disorder, involving harmful patterns (comparable to risky or problematic use), whereas moderate to severe cases would meet the full criteria for a behavioral addiction.

### Study objectives

This paper presents the development of the Addictive Daydreaming Scales, available in both full and short versions. Study 1 evaluated content validity and identified the most diagnostically relevant items for the screening version through expert ratings. In Study 2, both versions were validated by comparing their scores with participants’ self-assessment of problematic fantasizing, performance on the Polish Maladaptive Daydreaming Scale (PMDS-16 and PMDS-5), and quality-of-life measures in the social and psychological domains. Preliminary cutoff scores were proposed and examined to identify individuals (1) at risk for maladaptive daydreaming, (2) with moderate addictive daydreaming, and (3) with severe addictive daydreaming.

We expected that:

H1. ADS scores will be higher in the mixed-clinical group than in the non-clinical group.
H2 Self-reported maladaptive daydreamers score higher on the ADS than others.
H2A & H2B. ADS scores will correlate positively with (H2A) the self-reported negative impact of daydreaming and (H2B) PMDS scores.
H3 & H4. ADS scores will correlate negatively with age (H3) and with social and psychological quality of life domains (H4).
H4A. Participants scoring above ADS cut-offs will report lower social and psychological quality of life than those below.

#### Study 1: developing the addictive daydreaming scale

To develop the items for the Addictive Daydreaming Scale, a team of six clinicians with experience in maladaptive daydreaming convened to discuss its addictive characteristics. Each expert independently generated items that reflected indicators of addictive daydreaming, including impaired control over the frequency, duration, or cessation of daydreaming; the prioritization of daydreaming over daily responsibilities and interests; and the persistence or escalation of the behavior despite negative consequences. They also proposed items capturing additional clinically relevant features, such as the use of fantasy for emotional regulation or the experience of excitement associated with daydreaming. All items were subsequently reviewed in a group setting. Through consensus, duplicate statements were eliminated, resulting in a unified pool of 22 items. Response options were developed to reflect the intensity or frequency of each symptom. Finally, the preliminary version of the scale was reviewed by six individuals with lived experience of maladaptive daydreaming to ensure clarity, relevance, and comprehensibility.

To develop a brief ADS for screening, another set of 14 experts—including psychologists, psychiatrists, and psychotherapists familiar with the syndrome—completed an anonymous Qualtrics survey. Participants were asked to review the proposed criteria for daydreaming disorder and evaluate each of the 22 items by responding to the following question: *To what extent does each of these questions relate to the core diagnostic criteria of addictive daydreaming?* Fourteen experts completed the survey and rated all 22 ADS items using a 5-point Likert scale (1 = Not at all, 2 = Slightly, 3 = Moderately, 4 = A lot, 5 = Extremely). The highest mean ratings (≥ 4.79) were recorded for items #14, #01, #16, #22, and #17 (see **Table 1**). These items were selected for inclusion in the short version of the instrument (ADS-5). Seventeen items achieved an average rating of 3.5 or higher, indicating strong content validity. Even the lowest-rated items averaged around 3, suggesting that the item pool did not include irrelevant questions. Because the goal of the short form was clinical screening rather than item reduction by statistical redundancy, the five highest-rated items were retained even if not all were included in the final 20-item version. This ensured maximal content and face validity for identifying core addictive features.

**Table 1:** Descriptive statistics for assessing the diagnosticity of ADS-22 items by 14 experts.

ADS item	Mean	St. error	SD	Min	Max
ADS-14 Fantasizing prevented me from completing important tasks (e.g., school or work).	4.93	.071	.267	4	5
ADS-01 My daydreaming got out of control and I found it difficult to stop imagining stories.	4.86	.097	.363	4	5
ADS-16 I preferred to remain in my inner world rather than make new friends or socialize with others.	4.86	.143	.535	3	5
ADS-22 Despite my efforts and desire to focus on something else, I couldn’t resist immersing myself in fantasy worlds.	4.79	.155	.579	3	5
ADS-17 I realized that I was escaping into the fantasy world instead of taking up challenges or engaging in various life experiences.	4.79	.214	.802	2	5
ADS-20 I would return to the fantasy world, even if there were periods when I managed to refrain from daydreaming.	4.71	.125	.469	4	5
ADS-10 I felt dissatisfied or irritated when something distracted me and prevented me from playing out various scenarios in my head.	4.71	.163	.611	3	5
ADS-15 I forgot about important things or missed deadlines because I was absorbed in fantasizing.	4.64	.169	.633	3	5
ADS-11 I was looking for an opportunity to withdraw from any situation that made it difficult for me to daydream.	4.57	.137	.514	4	5
ADS-03 I was seeking opportunities to detach from current matters and immerse myself in the fantasy world.	4.57	.137	.514	4	5
ADS-21 Despite trying to control my thoughts, I would return to daydreaming.	4.57	.173	.646	3	5
ADS-12 When something in the real world interrupted my daydreaming, I tried to return to the fantasy world as quickly as possible.	4.36	.169	.633	3	5
ADS-08 I spent a lot of time (even several hours) imagining various stories and scenarios.	4.21	.300	1.122	2	5
ADS-09 I was so absorbed in the imagined scenarios that I lost track of long stretches of time.	4.14	.312	1.167	2	5
ADS-07 I felt that the greatest satisfaction would come from immersing myself in my imaginary world.	3.86	.254	.949	2	5
ADS-19 I focused so much on my own fantasies that I stopped paying attention to things that were happening around me.	3.57	.441	1.651	1	5
ADS-02 I was absorbed in my own dreams, where I created and played out various scenarios.	3.50	.429	1.605	1	5
ADS-04 Thanks to my fantasies, I was able to feel better.	3.21	.422	1.578	1	5
ADS-18 I was not sure whether something happened for real or just in my imagination.	3.21	.447	1.672	1	5
ADS-05 The scenarios played out in my head evoked excitement, a sense of empowerment, or triumph in me.	3.07	.355	1.328	1	5
ADS-13 I have heard from others that I am daydreaming instead of focusing on my responsibilities.	2.93	.412	1.542	1	5
ADS-06 I felt pleasure imagining things that didn’t really happen.	2.93	.385	1.439	1	5

#### Study 2: validation of the ADS full and short versions

## Method

### Participants

The non-clinical group (N = 231, 171 women, 60 men, M_age_ = 32.70, SD_age_ = 10.711) was recruited using university mailing lists and social media. Invitations were sent to students of three different universities and Facebook users interested in: yoga, relaxation, sports, well-being, films, and gaming. The mixed-clinical group consisted of people who were currently under psychological or psychiatric care due to various mental illnesses and disorders (N = 182, 136 women, 45 men, M_age_ = 33.25, SD_age_ = 9.853). They responded to announcements about the study or were referred by mental health professionals. The groups did not differ significantly in terms of gender or age. All participants registered using a web application containing an electronic version of the questionnaires. They could participate anonymously, but could also provide contact information (telephone or email) if they wished to receive feedback about their scores.

### Measures

#### Participant’s profile

A brief demographic and clinical survey assessing age, gender, education, marital status, current or past use of mental health services, psychiatric diagnoses (if known).

#### Addictive daydreaming scale

Participants completed the preliminary 22-item version of the scale, rating each statement on a 6-point frequency scale (0 = Not at all; 1 = Once in the last month; 2 = 2–3 times in the last month; 3 = On average once a week in the last month; 4 = More than once a week in the last month; 5 = Every day in the last month). The total score is computed as the sum of item ratings. Following psychometric analyses, two items were removed (item 18 for low item–total correlation; item 22 for redundancy with item 21), yielding the 20-item version (ADS-20). The short form (ADS-5) was derived from expert ratings of diagnostic relevance (Study 1). Although item 22 was excluded from the 20-item version due to redundancy, it was retained in the short form (ADS-5) because experts identified it as one of the most diagnostically central indicators of addictive daydreaming.

#### Polish maladaptive daydreaming scale

The PMDS-16 is a validated 16-item Polish adaptation of the original MDS-16, measuring the severity of maladaptive daydreaming over the past month. Items are scored on an 11-point scale (0%–100%), with the total score calculated as the mean. A cutoff score of 42 was used to indicate probable MD (22). The PMDS-5 is a 5-item short form used for screening, with the same response format and cutoff.

#### WHOQOL-BREF (psychological and social domains)

Selected items from the Polish version of the WHOQOL-BREF (34) were used to assess quality of life in the psychological (6 items) and social (3 items) domains. Items were averaged within each domain and multiplied by four to match WHOQOL-100 scaling.

#### Daydreaming’s impact (self-assessment)

Participants rated the perceived negative impact of their daydreaming using a single item: “*To what extent does your involvement in a fantasy world negatively affect your professional or private life?*” Responses were given on a 5-point Likert scale (1 = Not at all, 2 = Slightly, 3 = Moderately, 4 = A lot, 5 = Extremely). Scores of 4 or 5 were interpreted as indicating significant impairment.

### Procedure

Following ethical approval from the SWPS University Research Ethics Board, study information was published on a dedicated website and distributed via university mailing lists, social media, and printed materials provided by mental health professionals. Participants accessed an online platform where they reviewed the study aims, procedures, confidentiality assurances, and their rights as participants. Informed consent was obtained electronically. Participants then completed a fixed sequence of self-report measures presented in the following order: Participant’s profile, the Addictive Daydreaming Scale (ADS), the Polish Maladaptive Daydreaming Scale (PMDS-16), the WHOQOL-BREF (psychological and social domains), and the Daydreaming’s Impact Self-Assessment. Two weeks later, participants were automatically invited to complete a retest of the ADS. A total of 39 participants (66.67% women; M_age_ = 36.05; SD_age_ = 10.756, including 11 from the mixed-clinical group) completed the retest.

### Statistical analysis

Dimensionality of the ADS-20 and ADS-5 was evaluated using exploratory factor analysis (EFA) with direct Oblimin rotation and confirmatory factor analysis (CFA). Reliability was assessed via internal consistency (Cronbach’s alpha, McDonald’s omega) and test–retest stability (Spearman correlation, paired t-test, and Wilcoxon signed-rank test).

To assess criterion validity, mean ADS scores were compared across clinical and non-clinical groups and between participants reporting problematic (≥ 4) *vs*. non-problematic (≤ 3) daydreaming on the self-assessment item, using t-tests or Mann–Whitney U tests as appropriate. Due to non-normal score distributions (confirmed via Kolmogorov–Smirnov tests), non-parametric tests were applied where necessary. Chi-squared tests and Cramer’s V were used to compare the prevalence of elevated ADS scores across groups, based on established cutoffs.

Construct validity was examined through Spearman correlations between ADS scores and related measures: PMDS-16, PMDS-5, and the psychological and social domains of the WHOQOL-BREF. Additional group comparisons were conducted to evaluate differences in quality of life and maladaptive daydreaming severity between participants scoring above and below ADS cutoff points.

All analyses were performed using SPSS 28.0, except for EFA, which was conducted in JASP 0.19.3. The significance level was set at α = .05 (two-tailed). There were no missing data in ADS-22; completion of additional measures was optional.

### Ethics

The study was conducted in accordance with the Declaration of Helsinki and received approval from the Ethical Board at the SWPS University (Approval No: WKEB39/02/2017). All participants provided digitally an informed consent before participation. To ensure confidentiality their names were coded. The authors declare no conflicts of interest.

## Results

### Factor analysis and reliability of the ADS-20 and ADS-5

Based on item–total correlation analysis, item 18 was excluded from the ADS due to its weak correlation with the total score (r = .398, p <.001), and item 22 was removed due to redundancy with item 21 (r = .851, p <.001). However, because experts rated item 22 as highly clinically relevant, it was retained in the ADS-5 together with items 1, 14, 16, and 17.

**Figure 2:**
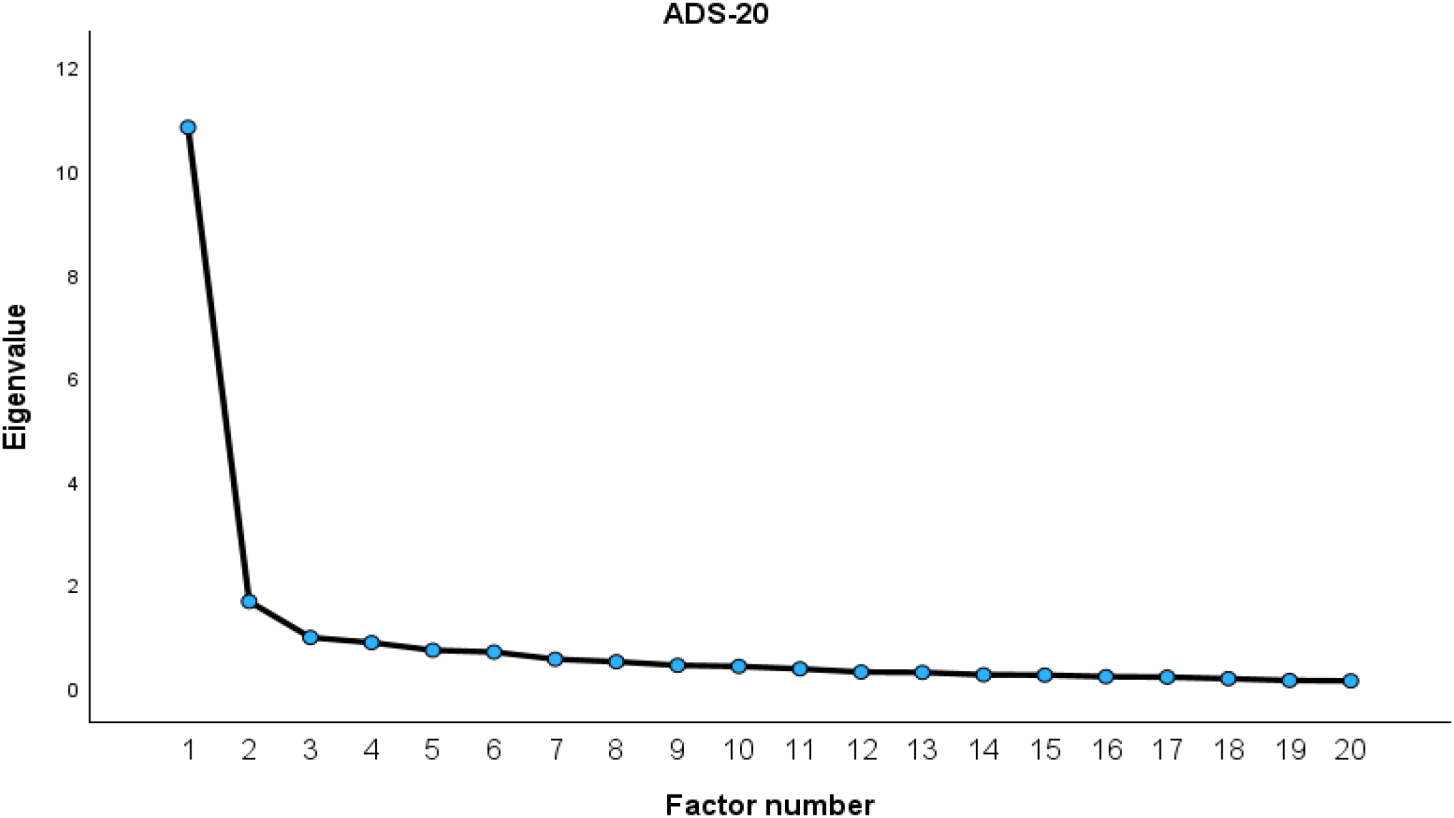
Scree plot for ADS-20.

Exploratory factor analysis (EFA) using principal axis factoring with direct Oblimin rotation was conducted on a randomly selected subsample (N = 203). For the ADS-20, both the scree plot (**Figure 2**) and Kaiser criterion supported a two-factor solution, which explained 62.5% of the total variance (Factor 1 = 54.2%, Factor 2 = 8.3%). Sampling adequacy was excellent (KMO = .934; Bartlett’s test: χ²(190) = 3106.87, *p* <.001).

**Figure 3:**
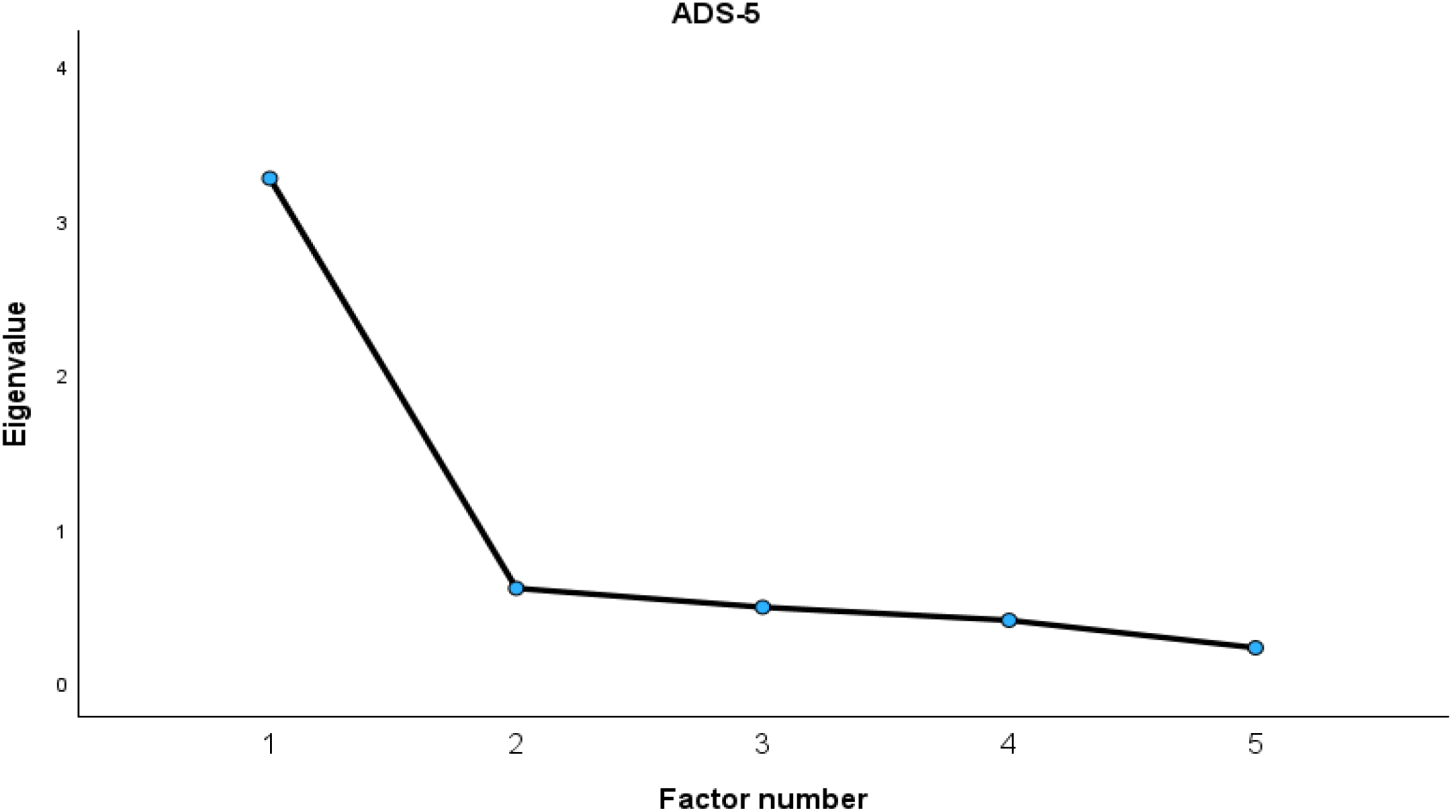
Scree plot for ADS-5.

For the ADS-5, both the scree plot (**Figure 3**) and the Kaiser criterion supported a one-factor solution. The analysis showed good sampling adequacy (KMO = .807; Bartlett’s test of sphericity: χ²(10) = 483.32, *p* <.001). This single-factor solution accounted for 65.4% of the total variance.

Subsequently, we calculated composite reliability (CR) and average variance extracted (AVE) for both the ADS-5 and ADS-20. For the ADS-5, CR = .904 and AVE = .654, indicating high construct reliability. For the ADS-20, we examined both a one-factor solution (CR = .959; AVE = .542) and a two-factor solution suggested by the EFA results. In the two-factor model, Factor 1 (Impairment; items 1, 3, 9–17, 19, 21) yielded CR = .904 and AVE = .460, and Factor 2 (Emotional regulation; items 2, 4–8, 20) yielded CR = .742 and AVE = .561. Although the AVE for Factor 1 in the EFA was slightly below.50, the CFA results showed improved estimates (AVE = .543 for Factor 1 and.704 for Factor 2). Exploratory and confirmatory factor analyses indicated that all items loaded strongly (>.50) on a single latent factor, and inter-factor correlations exceeded.85, suggesting substantial overlap between the two dimensions. Therefore, it seems reasonable to consider the ADS-20 as a unidimensional measure.

CFA for ADS-5 and ADS-20 was performed with other participants (N = 210) whose results were not included in EFA. We compared a 1-factor model with a 2-factor model for ADS_20 based on the rotated component matrix from EFA (see **Table 2**). The estimation of the fit of the alternative models was based on indicators contained in **Table 3**.

**Table 2:** Rotated component matrix for ADS-20.

Items	Factors	
	Impairment	Emotional regulation
ADS-04 Thanks to my fantasies, I was able to feel better.	-.122	**-.934**
ADS-08 I spent a lot of time (even several hours) imagining various stories and scenarios.	.399	**-.485**
ADS-13 I have heard from others that I am daydreaming instead of focusing on my responsibilities.	**.691**	.136
ADS-11 I was looking for an opportunity to withdraw from any situation that made it difficult for me to daydream.	**.601**	-.158
ADS-01 My daydreaming got out of control and I found it difficult to stop imagining stories.	**.591**	-.218
ADS-15 I forgot about important things or missed deadlines because I was absorbed in fantasizing.	**.951**	.261
ADS-07 I felt that the greatest satisfaction would come from immersing myself in my imaginary world.	.293	**-.608**
ADS-20 I would return to the fantasy world, even if there were periods when I managed to refrain from daydreaming.	.292	**-.588**
ADS-06 I felt pleasure imagining things that didn’t really happen.	-.070	**-.957**
ADS-02 I was absorbed in my own dreams, where I created and played out various scenarios.	.174	**-.778**
ADS-14 Fantasizing prevented me from completing important tasks (e.g., school or work).	**.812**	.095
ADS-16 I preferred to remain in my inner world rather than make new friends or socialize with others.	**.613**	-.145
ADS-03 I was seeking opportunities to detach from current matters and immerse myself in the fantasy world.	**.623**	-.242
ADS-05 The scenarios played out in my head evoked excitement, a sense of empowerment, or triumph in me.	.157	-**.762**
ADS-09 I was so absorbed in the imagined scenarios that I lost track of long stretches of time.	**.748**	-.127
ADS-10 I felt dissatisfied or irritated when something distracted me and prevented me from playing out various scenarios in my head.	**.488**	-.264
ADS-12 When something in the real world interrupted my daydreaming, I tried to return to the fantasy world as quickly as possible.	**.508**	-.354
ADS-21 Despite trying to control my thoughts, I would return to daydreaming.	**.637**	-.233
ADS-17 I realized that I was escaping into the fantasy world instead of taking up challenges or engaging in various life experiences.	**.648**	-.246
ADS-19 I focused so much on my own fantasies that I stopped paying attention to things that were happening around me.	**.767**	-.048

Extraction method: Principal axis factoring. Rotation: Direct Oblimin (Kaiser normalization). The rotation converged in 9 iterations. Boldface indicates primary factor loadings for item assignment to the respective factor.

**Table 3:** Confirmatory factor analysis results for ADS-20: a comparison of the fit of alternative factor models (N = 210).

Model	χ2	df	χ2/df	RMSEA (95% CI)	CFI	NFI	SRMR
1-factor	774.272	170	4.554	.130 (.121;.131)	.819	.781	.063
2-factor	546.234	169	3.232	.103 (.094;.113)	.887	.845	.054

2-factor model according to EFA results: F1 (items: 1, 3, 9-17, 19, 21) F2 (items 2, 4-8, 20).

RMSEA for both models is slightly (especially in the case of the 2-factor model) above the desired level 1, and χ2/df values exceed 3 (in the case of the 2-factor model, very slightly) (35). CFI for the 2-factor model is close to the recommended value of.09, also for the 1-factor solution is above.08. NFI for the 2-factor model is slightly lower than.9, and for the 1-factor model is close to.08. However, SRMR in all cases are distinctly below.08, which supports the admissibility of both models. From the clinical point of view, it is most important that the instrument enables measuring the level of AD accurately and reliably. Given this and the parsimony criterion, the total ADS-20 score was retained for subsequent analyses. CFA results for ADS-5 1-factor model mostly confirm fit of this model with good CFI, NFI, SRMR, and close to acceptable RMSEA (χ2(5) = 16.322, p = .050; CFI = .976; NFI:.966; RMSEA = 0.104 (95% CI:.050;.162); SRMR = .029).

The internal consistency for both ADS-20 (Cronbach’s Alpha = .958; McDonald’s Omega = .958) and ADS-5 (Cronbach’s Alpha = .862; McDonald’s Omega = .864) was high. Both ADS-20 (rho=.899, p<.001) and ADS-5 (rho=.861, p<.001) scores in baseline and the second measurement (N = 39) after a few weeks were highly correlated. ADS-20 scores in baseline (M = 31.513, SD = 29.752) and the second measurement (M = 29.667, SD = 25.387) did not differ significantly (t(N = 39) =-.986, p = .330; Z = -.809, p = .418). Analogously, in ADS-5 scores the first (M = 6.846, SD = 7.600) and second measurements (M = 7.679; SD = 7.975) were not different (t(N = 39) = .132, p = .896; Z = -.154, p = .878).

### Validation of the ADS-20 and ADS-5

To establish cut-off points for identifying addictive daydreaming, ROC curve analyses were conducted using self-reported negative impact of daydreaming on life (responses of “A lot” or “Extremely”) as the criterion. The ADS-20 demonstrated good discrimination accuracy (cut-off = 41.5, AUC = .813, 95% CI [.757,.869]), as did the ADS-5 (cut-off = 7.5, AUC = .848, 95% CI [.797,.900]).

Alternative cut-off points for moderate and severe MD were also tested, calculated as the number of items multiplied by response values of “3 – On average once a week” and “4 – More than once a week,” respectively, indicating moderate and severe levels of problematic daydreaming. When these thresholds were applied, the ADS-5 showed stronger performance than the ADS-20. However, ROC-derived cutoffs yielded higher specificity and accuracy for the ADS-20 (see **Table 4**).

**Table 4:** Addictive daydreaming, as assessed by the ADS-20, ADS-5, and self-assessment of negative impact daydreaming on life using alternative cutoff points.

ADS-20				ADS-5			
Diagnostic test	Daydreaming - negative life impact	Daydreaming - no life impact	Total	Diagnostic test	Daydreaming - negative life impact	Daydreaming - no life impact	Total
Positive (X *≥* 42)	51	67	118	Positive (X *≥* 8)	58	81	139
Negative (X < 42)	19	232	251	Negative (X < 8	12	218	230
Total	70	299	369	Total	70	299	369
Sensitivity (95%CI)	72.86% (60.90% to 82.80%)			Sensitivity (95%CI)	82.86% (71.97% to 90.82%)		
Specificity (95%CI)	77.59% (72.44% to 82.19%)			Specificity (95%CI)	72.91% (67.49% to 77.87%)		
PPV (95%CI)	43.22% (37.11% to 49.55%)			PPV (95%CI)	41.73% (36.63% to 47.01%)		
NPV (95%CI)	92.43 (89.22% to 94.74%)			NPV (95%CI)	94.78% (91.53% to 96.83%)		
Accuracy (95%CI)	76.69% (72.04% to 80.91%)			Accuracy (95%CI)	74.80% (70.04% to 79.15)		
Diagnostic test	Daydreaming - negative life impact	Daydreaming - no life impact	Total	Diagnostic test	Daydreaming - negative life impact	Daydreaming - no life impact	Total
Positive (X *≥* 60)	29	25	54	Positive (X ≥ 15)	34	15	49
Negative (X < 60)	41	274	315	Negative (X < 15)	36	284	320
Total	70	299	369	Total	70	299	369
Sensitivity (95%CI)	41.43% (29.77% to 53.83%)			Sensitivity (95%CI)	48.75 (36.44% to 60.83%)		
Specificity (95%CI)	91.64% (87.90% to 94.52%)			Specificity (95%CI)	94.98% (91.86% to 97.17%)		
PPV (95%CI)	53.70% (42.09% to 64.93%)			PPV (95%CI)	69.39% (56.69% to 79.69%)		
NPV (95%CI)	86.98% (84.55% to 89.09%)			NPV (95%CI)	88.75% (86.25% to 90.84%)		
Accuracy (95%CI)	82.11% (77.81% to 85.89%)			Accuracy (95%CI)	86.18% (82.23% to 89.53%)		
Diagnostic test	Daydreaming - negative life impact	Daydreaming - no life impact	Total	Diagnostic test	Daydreaming - negative life impact	Daydreaming - no life impact	Total
Positive (X *≥* 80)	10	4	14	Positive (X *≥* 20)	17	3	20
Negative (X < 80)	60	295	355	Negative (X < 20	53	296	349
Total	70	299	369	Total	70	299	369
Sensitivity (95%CI)	14.29% (7.07% to 24.71%)			Sensitivity (95%CI)	24.29% (14.83% to 36.01%)		
Specificity (95%CI)	98.66% (96.61% to 99.63%)			Specificity (95%CI)	99.00% (97.10% to 99.79%)		
PPV (95%CI)	71.43% (44.68% to 88.56%)			PPV (95%CI)	85.00% (63.07% to 94.95%)		
NPV (95%CI)	83.10% (81.70% to 84.41%)			NPV (95%CI)	84.81% (83.02% to 86.45%)		
Accuracy (95%CI)	82.66% (78.40% to 86.38%)			Accuracy (95%CI)	84.82% (80.75% to 88.33%)		

### Criterion validity examination

Group comparisons confirmed that scores on both ADS-20 and ADS-5 were significantly higher in the mixed-clinical group than in the non-clinical group (see **Tables 5**, **6**), confirming H1. Using the ROC-based thresholds, a significantly greater proportion of participants in the clinical group scored above the cut-off for both ADS-20 (χ²(1, 413) = 5.30, *p* = .021, Cramer’s V = .113) and ADS-5 (χ²(1, 413) = 7.25, *p* = .007, Cramer’s V = .132). Differences at higher, alternative cut-offs (e.g., ×3 and ×4 thresholds) were not statistically significant.

**Table 5:** Descriptive statistics for ADS-20 and ADS-5 for the mixed-clinical and non-clinical groups.

Group	N	Mean	SD	Mean rank	Sum of rank	% above 7.5	% above 14.5	% above 19.5
ADS-5								
Non-clinical	231	7,418	6,813	188,88	43632,00	31.6%	11.3%	5.2%
Mixed-clinical	182	5,460	6,457	229,99	41859,00	44.5%	17.6%	7.7%
Total	413							
ADS-20								
Non-clinical	231	34,522	23,722	190,35	43971,00	27.3%	13.4%	4.3%
Mixed-clinical	182	27,909	24,505	228,13	41520,00	37.9%	16.5%	3.8%
Total	413							

**Table 6:** Independent samples t-Test and the Mann-Whitney U test for ADS-5 and ADS-20 in non-clinical (N = 231) and mixed-clinical (N = 182) groups.

Scale	Mean difference	Standard error of the mean	95% Confidence interval of the difference		t-test				Mann-Whitney U test		
			Lower	Upper	t	df	Sig (2-tailed)	d Cohens	U	Z	Sig (2-tailed)
ADS-5	1.959	.656	.670	3.248	2.987	411	.003	.296	16836,00	-3.515	<.001
ADS-20	6.613	2.395	1.905	11.321	2.761	411	.006	.274	17175.00	-3.194	<.001

As predicted in H2, those reporting a strong negative impact of daydreaming (scores 4 and 5 on the Self-assessment) had substantially higher mean scores on both ADS-20 and ADS-5 compared to others (see **Tables 7**, **8**). A significantly higher proportion of them also exceeded the ROC-based and alternative thresholds on both scales (all *p* <.001, Cramer’s V = .266–.503), indicating strong discriminatory power.

**Table 7:** Descriptive statistics for ADS-5 and ADS-20 by self-reported negative impact of daydreaming.

Negative impact of daydreaming	N	Mean	SD	Mean rank	Sum of rank	% above 7.5	% above 14.5	% above 19.5
ADS-5								
Yes	70	13.586	6.811	160,61	48023,50	82.9%	48.6%	24.3%
No	299	4.552	5.100	289,16	20241,50	27.1%	5%	1.0%
ADS-20								
Negative impact of daydreaming	N	Mean	SD	Mean rank	Sum of rank	% above 41.5	% above 59.5	% above 79.5
Yes	70	53.829	22.824	163,06	48755,00	72.9%	41.4%	14.3%
No	299	25.582	20.950	278,71	19510,00	22.4%	58.6%	1.3%

**Table 8:** Independent samples t-test and Mann-Whitney U test results for ADS-5 and ADS-20 scores among participants with (N = 70) and without (N = 299) self-reported negative impact of daydreaming.

Scale	Mean difference	Standard error of the mean	95% Confidence interval of the difference		t-test				Mann-Whitney U test		
			Lower	Upper	t	df	Sig (2-tailed)	d Cohens	U	Z	Sig (2-tailed)
ADS-5	-9.034	.866	-10.460	-7.608	-10.434	367	<.001	-1.654	3173.50	-9.173	<.001
ADS-20	-28.247	2.830	-33.812	-22.681	-9.981	367	<.001	-1.325	3905.00	-8.168	<.001

### ADS construct validity, associations with quality of life and age

Spearman correlations (see **Table 9**) revealed strong positive associations between ADS-20/ADS-5 and PMDS-16/PMDS-5 (confirming H2B); moderate positive correlations with self-assessed negative impact (confirming H2A); and moderate negative correlations with quality of life in the Psychological and Social Relationship domains (WHOQOL-BREF), as expected in H4. Notably, correlations between ADS scores and quality of life were stronger than those observed for PMDS scores, supporting the added utility of ADS in capturing functional impairment. Both ADS versions were also weakly, negatively correlated with age (confirming H3).

**Table 9:** Descriptive statistics and Spearman correlations among ADS-20, ADS-5, PMDS-16, PMDS-5, WHOQOL-Bref psychological and social relation domains, self-assessment, and age.

Variables	Mean	SD	Skewness	Kurtosis	N	2	3	4	5	6	7	8
1. ADS-20	30.823	24.357	.635	-.582	413	.889**	.865**	.807**	.551**	-.526**	-.420**	-.373**
2. ADS-5	6.322	6.679	.954	-.064	413		.794**	.817**	.592**	-.571**	-.456**	-.279**
3. PMDS-16	32.360	22.366	.543	-.619	398			.907**	.591**	-.446**	-.324**	-.418**
4. PMDS-5	26.201	26.513	.886	-.287	398				.652**	-.492**	-.375**	-.324**
5. Self-assessment	2.23	1.224	.634	-.734	369					-.368**	-.297**	-.154*
6. WHOQO-Bref Psychological domain	12.602	3.073	-.263	-.569	369						.635**	.223**
7. WHOQO-Bref Social domain	12.669	3.507	-.387	-.482	369							.087
8. Age	32.94	10.333	.544	-.310	413							

**p<.001. *p<.01.

As predicted in H4A, participants scoring above ADS cut-offs reported significantly lower quality of life in both domains. Effect sizes were generally large and consistent across multiple cut-off points (ADS-5: *d* = .882–1.459; ADS-20: *d* = .770–1.036), with the strongest differences found at lower and mid-range thresholds (see **Table 10**).

**Table 10:** Descriptive statistics for WHOQOL-Bref psychological and social relationship domains by ADS-5 and ADS-20 scores below and above alternative cut-off points.

Group	N	Mean	SD	Mean rank	Sum of rank
WHOQOL-Bref psychological domain					
ADS-5 < 8	230	13.704	2.657	223.43	51390.00
ADS-5 ≥ 8	139	10.777	2.845	121.40	16875.00
ADS 20 < 42	251	13.519	2.747	217.06	54481.50
ADS-20 ≥ 42	18	10.650	2.817	116.81	13783.50
ADS-5 < 15	320	13.035	2.955	200.13	64041.00
ADS-5 ≥ 15	49	9.769	2.218	86.20	4224.00
ADS 20 < 60	315	13.024	2.959	199.55	62859.50
ADS-20 ≥ 60	54	10.136	2.539	100.10	5405.50
ADS-5 < 20	349	12.816	2.975	191.96	66993.00
ADS-5 ≥ 20	20	8.867	2.300	63.60	1272.50
ADS 20 < 80	355	12.710	3.061	188.93	67068.50
ADS-20 ≥ 80	14	9.857	1.942	85.46	1196.50
WHOQOL-Bref social relations domain					
ADS-5 < 8	230	13.762	3.085	218.23	510194.00
ADS-5 ≥ 8	139	10.859	3.422	130.01	18071.00
ADS 20 < 42	251	13.493	3.209	209.87	52676.50
ADS-20 ≥ 42	18	10.915	3.482	132.11	15588.50
ADS-5 < 15	320	13.063	3.305	195.89	62685.50
ADS-5 ≥ 15	49	10.095	3.732	113.87	5579.50
ADS 20 < 60	315	13.130	3.242	198.07	62390.50
ADS-20 ≥ 60	54	9.975	3.804	108.79	5874.50
ADS-5 < 20	349	12.932	3.341	191.93	66983.50
ADS-5 ≥ 20	20	8.067	3.193	64.08	1281.50
ADS 20 < 80	355	12.770	3.449	187.82	66677.00
ADS-20 ≥ 80	14	10.095	4.108	113.43	1588.00

## Discussion

The aim of this study was to develop and validate the Addictive Daydreaming Scale (ADS-20) and its short screening version (ADS-5) in a Polish sample, to assess MD within the behavioral addiction framework, to establish the psychometric properties of these tools, and to evaluate their clinical utility in detecting MD and differentiating levels of symptom severity.

To date, no existing instrument has assessed MD through the framework of behavioral addiction. Both the ADS-20 and ADS-5 demonstrated high reliability and strong content validity. The ADS-5 was intentionally derived from expert judgment rather than statistical trimming, as the aim was to develop a clinically sensitive screening tool rather than a psychometrically redundant subset of the full scale. This approach explains minor differences in item composition between the two versions and ensured maximal content validity for identifying core addictive features. Notably, both scales showed stronger correlations with the psychological and social domains of quality of life compared to the PMDS-16, suggesting that they may more accurately reflect the functional impairments and compulsive aspects characteristic of behavioral addictions. The response format of both versions, capturing the frequency of symptoms over the past four weeks, enhances their clinical utility—particularly for monitoring treatment progress.

Using a screening tool and asking direct questions about excessive daydreaming is especially important because individuals with MD often experience shame and may not disclose their difficulties unless specifically inquired about (7, 36). Identifying daydreaming disorder as an isolated symptom (26) and recognizing its potential to interfere with ongoing treatment of comorbid conditions is crucial (16, 37).

While EFA of the ADS-20 revealed a two-factor solution – distinguishing between emotional regulation (e.g., fantasy used for mood enhancement) and functional impairment (e.g., loss of control, interference with daily life) – CFA supported both one- and two-factor models. The two factors were strongly correlated, and the unidimensional solution was retained for its parsimony, reliability, and clinical usefulness, particularly in screening and treatment contexts. Conceptually, this supports treating addictive daydreaming as a cohesive construct that integrates affective, cognitive, and compulsive dimensions, in line with established behavioral addiction frameworks.

Preliminary findings suggest that a cut-off score of 42 on the ADS-20 and 8 on the ADS-5 may be indicative of MD. However, further research is needed to establish empirically validated thresholds corresponding to mild, moderate, and severe levels of daydreaming disorder.

The present findings also contribute to the ongoing debate concerning the psychiatric classification of MD (26). Although MD involves absorption, the strong associations between ADS scores and impairment in psychological and social functioning, point to a compulsive, reward-driven behavioral pattern more aligned with addiction models. Importantly, the ADS emphasizes features such as impaired control, prioritization of daydreaming over other activities, and continued use despite negative consequences – all of which are hallmark indicators of behavioral addiction.

Recent evidence further highlights the addictive qualities of maladaptive daydreaming. For example, Theodor-Katz and Soffer-Dudek (20) describe MD as an ‘excessive, addictive immersion’ in fantasies, marked by a compulsive yearning to withdraw into an inner world for emotional compensation. Although they conceptualize MD primarily as a dissociative disorder, their phenomenological description resonates strongly with addiction models. These features parallel core components of behavioral addictions, including craving, mood modification, and conflict, thereby strengthening the case for considering MD within an addiction framework.

### Limitations and further directions

This study has several limitations, primarily related to sample characteristics. Due to the lack of clinically diverse comparison groups, the discriminant validity of the instruments could not be assessed. Moreover, the relatively small number of participants scoring in the higher symptom range (i.e., ADS > 80) limited our ability to establish empirically grounded severity thresholds. The absence of structured clinical interviews to confirm whether these individuals met the proposed diagnostic criteria for daydreaming disorder also constrained interpretation. As a result, the determination of clinically meaningful cut-off scores remains preliminary.

The recruitment strategy – framed around daydreaming as a coping strategy – may have attracted participants already inclined toward fantasy, potentially inflating rates of reported symptoms. Thus, future research should examine the prevalence of addictive daydreaming using more representative samples. This would also allow further exploration of the observed negative correlation between age and symptom severity, consistent with previous studies (12, 36, 38), and could inform age-specific cut-off scores. Although there was an overrepresentation of female participants—a pattern common in psychological research and also observed in prior MD studies (38–40) – no gender effect was identified in the present data.

Further research should aim to test the validity and utility of the ADS-20 and ADS-5 across different languages and cultural contexts. Cross-cultural replication is essential for determining whether addictive daydreaming manifests similarly across sociocultural environments. Longitudinal studies are also necessary to assess the stability of symptoms over time and to evaluate the predictive validity of ADS scores in relation to functional impairment, relapse, or treatment response. In addition, developing a structured, in-depth clinical interview specifically targeting the symptoms of daydreaming disorder from a behavioral addiction perspective and consistent with the proposed diagnostic criteria is a critical next step, as no such tool currently exists. Although the Maladaptive Daydreaming Interview (36) is available, it was designed to assess maladaptive daydreaming as a dissociative phenomenon rather than as a behavioral addiction. Such an instrument would enable clinicians to confirm or rule out diagnoses with greater accuracy, thereby improving diagnostic procedures and research reliability. Validation of the ADS-20 and ADS-5 should include analyses involving individuals with formally assessed diagnoses of daydreaming disorder, allowing for the refinement of cut-off points and clarification of mild, moderate, and severe cases (see: 12). Finally, neurobiological investigations are needed to uncover the underlying mechanisms of addictive daydreaming and to clarify its relationship with other behavioral addictions. A deeper understanding of these processes could inform the development of more targeted treatment strategies and broaden the conceptualization of compulsive internal experiences.

## Conclusion

The ADS-20 and ADS-5 are reliable and valid instruments for identifying and assessing MD within an addictive paradigm. By measuring both frequency and associated impairment, they offer a clinically useful framework for screening, severity grading, and tracking changes over time. Their stronger associations with quality-of-life deficits than existing MD measures support conceptualizing this syndrome as a behavioral addiction. Further validation with structured clinical interviews and cross-cultural research is needed to refine diagnostic thresholds and confirm their applicability in diverse contexts.

## Data Availability

The datasets presented in this study can be found in online repositories. The names of the repository/repositories and accession number(s) can be found below: https://osf.io/w9rm4/?view_only=dd334d9c093f483eb196d5af0e42e43c.

## Supplementary material

Supplementary Data Sheet 1 = Appendix 1 Proposed Criteria for daydreaming disorder.
