# Supplementary material for: Introducing the addictive daydreaming scale: development and Polish validation of the ADS-20 and ADS-5
Source: Front Psychiatry. 2025 Dec 15;16:1702416. doi: 10.3389/fpsyt.2025.1702416 (PMC12745272; doi:10.3389/fpsyt.2025.1702416)
Supplement: Supplementary Data Sheet 1 = Appendix 1 — Proposed Criteria for daydreaming disorder. [file DataSheet1.pdf]

Appendix 1

Proposed criteria for Daydreaming disorder

Daydreaming disorder is characterised by a persistent or recurrent pattern of maladaptive daydreaming, manifested by **all** of the following:

Criterion A — Impaired Control

Persistent or recurrent difficulty regulating daydreaming, reflected by **any** of the following:

1. **Failed attempts to limit** the onset, duration, frequency, or termination of daydreaming, or
2. **Marked inability to regulate daydreaming** when regulation would normally be expected, even when the individual does *not* actively try to resist (e.g., automatic engagement, habitual surrender, avoidance of testing control, low insight).

Control may be passively impaired (indulgence) or actively impaired (failed attempts).

Criterion B — Increasing Priority Given to Daydreaming

Daydreaming progressively takes precedence over other activities and responsibilities, or repeatedly replaces required tasks, social interactions, self-care, or goal-directed behaviour. This includes behavioural or mental reallocation of time, attention, and motivation toward fantasising.

Criterion C — Continuation Despite Harm

Daydreaming continues despite harm, defined as either:

1. **significant distress** (e.g., shame, guilt, frustration, fear of losing control),

or

2. **impairment** in personal, social, educational, occupational, or other important areas of functioning.

Distress OR impairment is sufficient. Insight may vary; individuals may continue despite harm even if they do not fully recognise it.

Duration

Symptoms present for **≥12 months**, unless severity is high and all criteria are met in a shorter period.

Severity specifiers

Operationalisation of severity is underway and currently being validated.

Boundary with Normality (Threshold):

1. Daydreaming Disorder should not be diagnosed solely based on frequent or prolonged daydreaming in the absence of the disorder's other core features, such as distress, functional impairment, or loss of control. Repetitive daydreaming, even when highly immersive, is not inherently pathological.
2. Many individuals engage in daydreaming as a normative mental activity. For example, people may fantasise about romantic relationships, social interactions, career success, or imaginary adventures. Such daydreams often serve adaptive functions — helping individuals plan future scenarios, rehearse conversations, explore identity, regulate mood, or cope with boredom. These fantasies are typically under voluntary control, can be paused or stopped when needed, and do not interfere with daily responsibilities or social functioning.
3. Daydreaming may also be integrated into social or creative activities, such as collaborative storytelling, writing fiction, planning role-playing games, or engaging in world-building with others. These behaviors, even when highly imaginative or absorbing, do not in themselves constitute a mental disorder.
4. High frequency or long duration of daydreaming — especially in adolescents or in contexts with ample unstructured time — is not sufficient for diagnosis unless accompanied by additional hallmark features such as 1) marked subjective distress related to the daydreaming; 2) difficulty controlling or limiting the activity despite intentions to do so; 3) significant interference with academic, occupational, or interpersonal functioning.

Cultural, subcultural, and peer-group norms must also be considered when evaluating whether daydreaming activity is deviant or impairing. For example, immersive fantasy engagement may be culturally normative in certain artistic, gaming, or neurodiverse communities. Regardless of the social contributions to the activity, the diagnosis of Daydreaming Disorder may still be applied if all diagnostic requirements are met. Daydreaming Disorder should also be distinguished from normal mind-wandering, rumination or intrusive thoughts.

Additional clinical features:

- If symptoms and consequences of compulsive daydreaming behaviour are severe (e.g., daydreaming behaviours persist for many hours a day or have major effects on functioning or health) and all other diagnostic requirements are met, it may be appropriate to assign a diagnosis of Daydreaming Disorder following a period that is briefer than 12 months (e.g., 6 months).
- Although daydreaming can provide temporary emotional relief—for instance, by boosting self-esteem, excitement, or feelings of importance and appreciation—individuals with Daydreaming Disorder may make numerous unsuccessful attempts to control or significantly reduce their daydreaming, whether self-initiated or externally imposed.
- Individuals with Daydreaming Disorder may increase the duration or frequency of daydreaming activity over time or experience a need to engage in fantasy to maintain or exceed previous levels of excitement or to avoid boredom.

- Individuals with Daydreaming Disorder may feel annoyed when their daydreaming is interrupted by others or external circumstances and may actively seek opportunities to resume fantasising.
- Individuals with Daydreaming Disorder often experience urges or cravings to engage in fantasy during other activities.
- Daydreaming may be triggered or intensified by stereotypical movements (e.g., rocking, pacing) or music.
- Upon cessation or reduction of daydreaming activity, often imposed by other people or circumstances, individuals with Daydreaming Disorder may experience dysphoria and exhibit adversarial behaviour or verbal or physical aggression.
- Individuals with Daydreaming Disorder may exhibit substantial disruptions in diet, sleep, exercise and other health-related behaviours that can result in negative physical and mental health outcomes, particularly if there are very extended periods of daydreaming.
- Daydreaming Disorder can co-occur with personality disorders, autism spectrum disorders, dissociative identity disorder, attention deficit hyperactivity disorder, disorders due to substance use.
