# Supplementary material for: Introducing the addictive daydreaming scale: development and Polish validation of the ADS-20 and ADS-5
Source: Front Psychiatry. 2025 Dec 15;16:1702416. doi: 10.3389/fpsyt.2025.1702416 (PMC12745272; doi:10.3389/fpsyt.2025.1702416)
Supplement: Supplementary file 2 [file SupplementaryFile1.pdf]

Participant: ___________________________________ Date: _______________

ADS-20

For many people, daydreaming can be a way of coping with difficult emotions or experiences. The questions in this test inquire about your use of fantasy.

How often have you had the following experiences in the last month?

1. Thanks to my fantasies, I was able to feel better.

- ☐ Not at all
- ☐ Once in the last month
- ☐ 2-3 times in the last month
- ☐ On average once a week over the last month
- ☐ More than once a week in the last month
- ☐ Every day for the last month

2. I spent a lot of time (even several hours) imagining various stories and scenarios.

- ☐ Not at all
- ☐ Once in the last month
- ☐ 2-3 times in the last month
- ☐ On average once a week over the last month
- ☐ More than once a week in the last month
- ☐ Every day for the last month

3. I have heard from others that I am daydreaming instead of focusing on my responsibilities.

- ☐ Not at all
- ☐ Once in the last month
- ☐ 2-3 times in the last month
- ☐ On average once a week over the last month
- ☐ More than once a week in the last month
- ☐ Every day for the last month

4. I was looking for an opportunity to withdraw from any situation that made it difficult for me to daydream.

- ☐ Not at all
- ☐ Once in the last month
- ☐ 2-3 times in the last month
- ☐ On average once a week over the last month
- ☐ More than once a week in the last month
- ☐ Every day for the last month

Copyright by Pietkiewicz, Tomalski, & Hełka (2025)
www.e-psyche.eu

5. **My daydreaming got out of control and I found it difficult to stop imagining stories.**

- ☐ Not at all
- ☐ Once in the last month
- ☐ 2-3 times in the last month
- ☐ On average once a week over the last month
- ☐ More than once a week in the last month
- ☐ Every day for the last month

6. **I forgot about important things or missed deadlines because I was absorbed in fantasising.**

- ☐ Not at all
- ☐ Once in the last month
- ☐ 2-3 times in the last month
- ☐ On average once a week over the last month
- ☐ More than once a week in the last month
- ☐ Every day for the last month

7. **I felt that the greatest satisfaction would come from immersing myself in my imaginary world.**

- ☐ Not at all
- ☐ Once in the last month
- ☐ 2-3 times in the last month
- ☐ On average once a week over the last month
- ☐ More than once a week in the last month
- ☐ Every day for the last month

8. **I would return to the fantasy world, even if there were periods when I managed to refrain from daydreaming.**

- ☐ Not at all
- ☐ Once in the last month
- ☐ 2-3 times in the last month
- ☐ On average once a week over the last month
- ☐ More than once a week in the last month
- ☐ Every day for the last month

9. **I felt pleasure imagining things that didn't really happen.**

- ☐ Not at all
- ☐ Once in the last month
- ☐ 2-3 times in the last month
- ☐ On average once a week over the last month
- ☐ More than once a week in the last month
- ☐ Every day for the last month

Copyright by Pietkiewicz, Tomalski, & Hełka (2025)
www.e-psyche.eu

10. I was absorbed in my own dreams, where I created and played out various scenarios.

- ☐ Not at all
- ☐ Once in the last month
- ☐ 2-3 times in the last month
- ☐ On average once a week over the last month
- ☐ More than once a week in the last month
- ☐ Every day for the last month

11. Fantasizing prevented me from completing important tasks (e.g., school or work).

- ☐ Not at all
- ☐ Once in the last month
- ☐ 2-3 times in the last month
- ☐ On average once a week over the last month
- ☐ More than once a week in the last month
- ☐ Every day for the last month

12. I preferred to remain in my inner world rather than make new friends or socialise with others.

- ☐ Not at all
- ☐ Once in the last month
- ☐ 2-3 times in the last month
- ☐ On average once a week over the last month
- ☐ More than once a week in the last month
- ☐ Every day for the last month

13. I was seeking opportunities to detach from current matters and immerse myself in the fantasy world.

- ☐ Not at all
- ☐ Once in the last month
- ☐ 2-3 times in the last month
- ☐ On average once a week over the last month
- ☐ More than once a week in the last month
- ☐ Every day for the last month

14. The scenarios played out in my head evoked excitement, a sense of empowerment, or triumph in me.

- ☐ Not at all
- ☐ Once in the last month
- ☐ 2-3 times in the last month
- ☐ On average once a week over the last month
- ☐ More than once a week in the last month
- ☐ Every day for the last month

Copyright by Pietkiewicz, Tomalski, & Hełka (2025)
www.e-psyche.eu

15. **I was so absorbed in the imagined scenarios that I lost track of long stretches of time.**

- Not at all
- Once in the last month
- 2-3 times in the last month
- On average once a week over the last month
- More than once a week in the last month
- Every day for the last month

16. **I felt dissatisfied or irritated when something distracted me and prevented me from playing out various scenarios in my head.**

- Not at all
- Once in the last month
- 2-3 times in the last month
- On average once a week over the last month
- More than once a week in the last month
- Every day for the last month

17. **When something in the real world interrupted my daydreaming, I tried to return to the fantasy world as quickly as possible.**

- Not at all
- Once in the last month
- 2-3 times in the last month
- On average once a week over the last month
- More than once a week in the last month
- Every day for the last month

18. **Despite trying to control my thoughts, I would return to daydreaming.**

- Not at all
- Once in the last month
- 2-3 times in the last month
- On average once a week over the last month
- More than once a week in the last month
- Every day for the last month

19. **I realised that I was escaping into the fantasy world instead of taking up challenges or engaging in various life experiences.**

- Not at all
- Once in the last month
- 2-3 times in the last month
- On average once a week over the last month
- More than once a week in the last month
- Every day for the last month

Copyright by Pietkiewicz, Tomalski, & Hełka (2025)
www.e-psyche.eu
e-psyche BADANIA

20. I focused so much on my own fantasies that I stopped paying attention to things that were happening around me.

- ☐ Not at all
- ☐ Once in the last month
- ☐ 2-3 times in the last month
- ☐ On average once a week over the last month
- ☐ More than once a week in the last month
- ☐ Every day for the last month

Copyright by Pietkiewicz, Tomalski, & Hełka (2025)
www.e-psyche.eu
