# Supplementary material for: Introducing the addictive daydreaming scale: development and Polish validation of the ADS-20 and ADS-5
Source: Front Psychiatry. 2025 Dec 15;16:1702416. doi: 10.3389/fpsyt.2025.1702416 (PMC12745272; doi:10.3389/fpsyt.2025.1702416)
Supplement: Supplementary file 3 [file SupplementaryFile2.pdf]

Participant: ____________________________ Date: ____________

ADS-5

For many people, daydreaming can be a way of coping with difficult emotions or experiences. The questions in this test inquire about your use of fantasy.

How often have you had the following experiences in the last month?

1. My daydreaming got out of control and I found it difficult to stop imagining stories.

- ☐ Not at all
- ☐ Once in the last month
- ☐ 2-3 times in the last month
- ☐ On average once a week over the last month
- ☐ More than once a week in the last month
- ☐ Every day for the last month

2. Fantasizing prevented me from completing important tasks (e.g., school or work).

- ☐ Not at all
- ☐ Once in the last month
- ☐ 2-3 times in the last month
- ☐ On average once a week over the last month
- ☐ More than once a week in the last month
- ☐ Every day for the last month

3. I preferred to remain in my inner world rather than make new friends or socialise with others.

- ☐ Not at all
- ☐ Once in the last month
- ☐ 2-3 times in the last month
- ☐ On average once a week over the last month
- ☐ More than once a week in the last month
- ☐ Every day for the last month

Copyright by Pietkiewicz, Tomalski, & Hełka (2025)
www.e-psyche.eu

4. **Despite my efforts and desire to focus on something else, I couldn't resist immersing myself in fantasy worlds.**

- ☐ Not at all
- ☐ Once in the last month
- ☐ 2-3 times in the last month
- ☐ On average once a week over the last month
- ☐ More than once a week in the last month
- ☐ Every day for the last month

5. **I realised that I was escaping into the fantasy world instead of taking up challenges or engaging in various life experiences.**

- ☐ Not at all
- ☐ Once in the last month
- ☐ 2-3 times in the last month
- ☐ On average once a week over the last month
- ☐ More than once a week in the last month
- ☐ Every day for the last month

Copyright by Pietkiewicz, Tomalski, & Hełka (2025)
www.e-psyche.eu
